# The Relationship Between Self-Reported Misophonia Symptoms and Auditory Aversive Generalization Leaning: A Preliminary Report

**DOI:** 10.3389/fnins.2022.899476

**Published:** 2022-06-23

**Authors:** Richard T. Ward, Faith E. Gilbert, Jourdan Pouliot, Payton Chiasson, Skylar McIlvanie, Caitlin Traiser, Kierstin Riels, Ryan Mears, Andreas Keil

**Affiliations:** ^1^Center for the Study of Emotion and Attention, University of Florida, Gainesville, FL, United States; ^2^Department of Psychology, University of Florida, Gainesville, FL, United States

**Keywords:** Misophonia, aversive auditory conditioning, generalization learning, sharpened tuning, valence, arousal

## Abstract

Misophonia is characterized by excessive aversive reactions to specific “trigger” sounds. Although this disorder is increasingly recognized in the literature, its etiological mechanisms and maintaining factors are currently unclear. Several etiological models propose a role of Pavlovian conditioning, an associative learning process heavily researched in similar fear and anxiety-related disorders. In addition, generalization of learned associations has been noted as a potential causal or contributory factor. Building upon this framework, we hypothesized that Misophonia symptoms arise as a consequence of overgeneralized associative learning, in which aversive responses to a noxious event also occur in response to similar events. Alternatively, heightened discrimination between conditioned threat and safety cues may be present in participants high in Misophonia symptoms, as predicted by associative learning models of Misophonia. This preliminary report (*n* = 34) examines auditory generalization learning using self-reported behavioral (i.e., valence and arousal ratings) and EEG alpha power reduction. Participants listened to three sine tones differing in pitch, with one pitch (i.e., CS+) paired with an aversive loud white noise blast, prompting aversive Pavlovian generalization learning. We assessed the extent to which overgeneralization versus heightened discrimination learning is associated with self-reported Misophonia symptoms, by comparing aversive responses to the CS+ and other tones similar in pitch. Behaviorally, all participants learned the contingencies between CS+ and noxious noise, with individuals endorsing elevated Misophonia showing heightened aversive sensitivity to all stimuli, regardless of conditioning and independent of hyperacusis status. Across participants, parieto-occipital EEG alpha-band power reduction was most pronounced in response to the CS+ tone, and this difference was greater in those with self-reported Misophonia symptoms. The current preliminary findings do not support the notion that overgeneralization is a feature of self-reported emotional experience in Misophonia, but that heightened sensitivity and discrimination learning may be present at the neural level.

## Introduction

Individuals with Misophonia experience decreased tolerance and aversive responses to specific auditory “trigger” cues (Jastreboff and Jastreboff, 2001, 2015; Swedo et al., 2022). Interest in this disorder has been steadily growing over the past years, given its association with adverse outcomes and comorbidity with other mental health disorders (Schröder et al., 2013; Wu et al., 2014; Cavanna and Seri, 2015; Webber and Storch, 2015; Zhou et al., 2017; Brout et al., 2018; Erfanian et al., 2019; Porcaro et al., 2019; Jager et al., 2020). However, there are still limited data regarding potential etiological mechanisms contributing to the emergence and maintenance of Misophonia. The present report presents initial data from an ongoing study of generalization learning, testing an extension of long-standing hypotheses in this area of research.

### Etiology of “Trigger” Cues

The auditory cues driving negative emotional reactions in Misophonia often include orofacial sounds (e.g., smacking lips, loud chewing, heavy breathing, sniffling, etc.) produced by other individuals (Jastreboff and Jastreboff, 2003, 2015; Edelstein et al., 2013; Schröder et al., 2013; Duddy and Oeding, 2014; Kumar et al., 2021; Vitoratou et al., 2021a; Swedo et al., 2022), regardless of the intensity of these sounds (Schröder et al., 2013; Jager et al., 2020; Swedo et al., 2022) or other alterations in physical properties of these auditory cues (Aazh et al., 2008, 2018; Edelstein et al., 2013; Schröder et al., 2013; Tyler et al., 2014; Jastreboff and Jastreboff, 2015; Potgieter et al., 2019; Swedo et al., 2022). The negative affective responses elicited by these cues comprise feelings of anxiety, fear, disgust, irritation, and anger directed at the individual eliciting them, and the avoidance of contexts or situations where these sounds may occur (Schröder et al., 2013; Cavanna and Seri, 2015; Potgieter et al., 2019; Swedo et al., 2022). This has led several to propose that these auditory cues hold some contextual value to individuals with Misophonia (Jastreboff and Jastreboff, 2003, 2015; Edelstein et al., 2013; Schröder et al., 2013; Duddy and Oeding, 2014), implying an etiological role for associative learning in the development of these cues. Taken together with the lack of altered physiology in Misophonic individuals, many have called for Misophonia to be treated as a mental health disorder separate from auditory perceptual disorders (Schröder et al., 2013; Taylor, 2017; Rouw and Erfanian, 2018; Jager et al., 2020; Swedo et al., 2022), with a primary emphasis on learning dynamics driving the development of symptomology. A recent consensual definition of Misophonia calls for the recognition of Misophonia as a disorder (Swedo et al., 2022).

Pavlovian conditioning has been considered as an etiological mechanism in Misophonia (Jastreboff and Jastreboff, 2001, 2002; Schröder et al., 2013; Dozier, 2015; Brout et al., 2018; Palumbo et al., 2018; Kumar et al., 2021). In one theoretical framework, Dozier (2015) hypothesized a two-step reflex process in response to auditory “trigger” cues (i.e., conditioned stimuli, CS+), with the cue inducing a physical muscular reflex, resulting in an emotional response (i.e., conditioned response, CR). Specifically, these auditory cues are hypothesized to be initially processed in the auditory cortex, which then provides input to the amygdala (Jastreboff and Jastreboff, 2001, 2002), consequentially activating the sympathetic nervous system and eliciting an emotional response (LeDoux, 2007, 2012). Complementing this notion, Kumar et al. (2021) theorized that other non-orofacial sounds may come to elicit adverse emotional reactions in individuals with Misophonia via associative learning, in which both an initial “trigger” cue is presented with a non-associated cue (Muller et al., 2018; Wiese et al., 2021). As such, Pavlovian conditioning is theorized to drive increased connectivity between limbic and autonomic sympathetic systems, resulting in the primary symptoms experienced in Misophonia in response to specific auditory “trigger” cues (Jastreboff and Hazell, 2004; Møller, 2011; Jastreboff and Jastreboff, 2013). Furthermore, these responses may over time generalize to other non-associated “trigger” cues (Dozier, 2015; Kumar et al., 2021).

### Generalization Learning

Aversive conditioning, a form of classical Pavlovian conditioning where a CS+ is learned to be associated with an unconditioned stimulus (i.e., US), has been applied extensively to study the development and maintenance of fear and anxiety disorders (Lissek et al., 2005; Reinhardt et al., 2010; Torrents-Rodas et al. (2013); Tinoco-González et al. (2015); Duits et al., 2015), which encompass co-occurring symptoms with Misophonia (Quek et al., 2018; Erfanian et al., 2019; Jager et al., 2020; Guetta et al., 2022). In addition to classical associative learning, some have proposed that individuals with Misophonia may come to experience heightened emotional responses to stimuli not related to orofacial sounds through separate associative and generalization learning processes (Dozier, 2015; Kumar et al., 2021). Generalization learning is an extension of simple differential aversive conditioning that allows for the assessment of how generalizable a conditioned response is to stimuli that are similar to a CS+ (Dunsmoor and Paz, 2015; Dymond et al., 2015; Struyf et al., 2015; Jasnow et al., 2017). In this process, a neutral stimulus is paired with a US, creating a CS+. In addition, other stimuli varying in physical similarity along a continuum (e.g., some closely resembling the CS+, while others may appear completely different) are presented but never paired with a US. This paradigm allows for the evaluation of conditioned responses to these non-paired stimuli, known as generalized stimuli (GS). Results from generalization learning have found that healthy control participants normally display a quadratic pattern of responses along this generalization gradient when measuring self-reported perceived risk of encountering a US (Lissek et al., 2010, 2014a), while those with anxiety-related disorders (e.g., Panic Disorder and Generalized Anxiety Disorder) display less of a decline from a CS+ to the nearest GS, indicative of overgeneralization in these clinical populations. Additional electrophysiological work with rodents found difference-of-Gaussian, or sharpened tuning, response patterns in auditory cortical cells (Bordi and LeDoux, 1994; Weinberger, 2007), while broadened-Gaussian patterns in cellular firing were observed in the medial geniculate portion of the thalamus (Edeline and Weinberger, 1992; Bordi and LeDoux, 1994) and a range of regions (e.g., insula, dorso- and ventromedial prefrontal cortex, etc.) in human neuroimaging work (Greenberg et al., 2013; Lissek et al., 2014b). These findings suggest that behavioral responses are likely to follow a Gaussian-like distribution (Ghirlanda and Enquist, 2003), while underlying neural mechanisms associated with these processes may yield either generalization or sharpened tuning response patterns.

**FIGURE 1:**
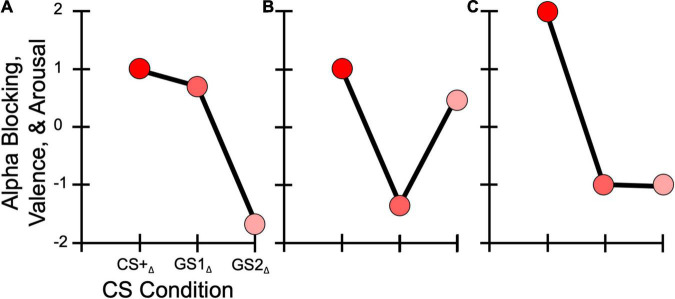
Hypothesized learning model response patterns. **(A)** Generalization, with the GS1 eliciting a greater response than the GS2. **(B)** Sharpening, in which GS1 has a decreased response compared to the GS2. **(C)** All-or-Nothing, where the GS1 and GS2 display similarly decreased responses relative to the CS+.

Synthesizing these findings, evidence of both generalization and sharpened tuning response patterns have been provided in human electroencephalography (EEG) research measuring visual sensory cortical responses (Müller et al., 1998; Wieser et al., 2016), and alpha-band power, a signal reflecting attentional processing (Deng et al., 2020) and heightened attentional engagement to a CS+ (Panitz et al., 2019). Specifically, parietal alpha power (Friedl and Keil, 2020, 2021) and steady-state visual evoked potentials (i.e., ssVEPs) displayed Gaussian distributions across the generalization gradient (McTeague et al., 2015), similar to neuroimaging work (Greenberg et al., 2013; Lissek et al., 2014b), and followed the generalization pattern shown in Figure 1A (Ghirlanda and Enquist, 2003). Parietal alpha-band activity (spectral power between 8 and 12 Hz) has been established as a robust index of stimulus saliency, linked to heightened attentional engagement with conditioned stimuli (Yin et al., 2018). Specifically, transient suppression of alpha power upon stimulus presentation has been taken to index the attentive engagement with conditioned threat cues, compared to safety cues or neutral cues (Panitz et al., 2019). The present study leveraged this effect as a manipulation check for successful conditioning, and examined its sensitivity to differences in Misophonia symptom status. During generalization learning, it is expected that as threat cues acquire increased task-relevance through conditioning, alpha power would show greater power reduction for the CS+ compared to the generalization stimuli. In contrast, ssVEPs recorded from occipital sites, commonly used to assess visual cortical perception, showed difference-of-Gaussian patterns (McTeague et al., 2015; Stegmann et al., 2020; Friedl and Keil, 2021). This suggests that non-sensory regions are likely to show Gaussian-like responses along a generalization gradient, while primary sensory cortices may yield sharpened tuning, both response patterns being adaptive, respectively, for optimizing perception (sharpening) and attentional orienting (generalization).

While previous work has investigated both autonomic (Edelstein et al., 2013; Kumar et al., 2017; Schröder et al., 2019) and neural responses to naturalistic auditory stimuli in individuals with Misophonia (Kumar et al., 2017, 2021; Schröder et al., 2019; Daniels et al., 2020), no study to our knowledge has examined how aversive learning processes contribute to auditory cues acquiring negative attributes. Furthermore, it is unclear whether Misophonic individuals display overgeneralized responses akin to what is commonly observed in fear and anxiety disorders (Lissek et al., 2005; Reinhardt et al., 2010; Duits et al., 2015). This is critical given the notion that Pavlovian conditioning serves as an etiological mechanism of symptomology in Misophonia (Jastreboff and Jastreboff, 2001, 2002; Schröder et al., 2013; Dozier, 2015; Brout et al., 2018; Palumbo et al., 2018; Kumar et al., 2021). Thus, we sought to address this gap by examining the mechanisms underlying aversive generalization learning in individuals with Misophonia (Dozier, 2015; Kumar et al., 2021). Participants completed an aversive generalization task consisting of an auditory sinewave tone presented at three different pitches over habituation and acquisition phases while EEG was recorded. In this design, one pitch served as the CS+, while the other two pitches differed in frequency from the CS+ and served as our GS. The inclusion of a habituation phase alongside the acquisition phase allowed us to examine changes over the course of learning relative to a baseline.

### Current Study

First, we aimed to examine the extent to which an auditory sinewave tone paired with a US (i.e., CS+) influenced ratings and EEG indices of attentional processing compared to other GS along a generalization continuum. We hypothesized that (H1) participants, regardless of Misophonia severity, would rate the CS+ tone as more aversive and arousing, and EEG signals reflecting greater attentional processing for the CS+ compared to the non-CS+ tones (i.e., the GS) during acquisition, in which the CS+ is paired with a loud noise US. This was assessed by self-reported behavioral ratings of valence and arousal, and stimulus-induced changes in parietal alpha-band power during two phases: an initial habituation (i.e., baseline) phase, in which no stimulus was paired with a US, and an acquisition phase. Furthermore, we expected (H2) the change in these dependent variables between the habituation and acquisition phases to be larger for the CS+ compared to the other GS presented.

Regarding self-reported symptoms of Misophonia, we investigated the impact symptom severity had on response patterns to the CS+ and GS. Specifically, we predicted that (H3A) participants endorsing greater Misophonia symptomology, measured through the Misophonia Symptom Scale (MSS; Wu et al., 2014), would show overgeneralized responses across the stimulus generalization gradient (Figure 1A), demonstrated via a Gaussian distribution pattern with greater responses to the CS+, similarly high responses to a similar GS (i.e., GS1), and low responses to a less similar GS (i.e., GS2). This would be reflected by better model fits for a generalization model compared to a sharpened tuning or all-or-nothing discrimination models. Specifically, model weights derived from the competing learning models (e.g., overgeneralization and sharpening) were applied to self-reported behavioral valence and arousal ratings, and parietal alpha-band power changes, with better model fit scores reflecting a stronger match between these dependent variables and the associated model. These hypotheses were guided by explicit models of generalization learning, as discussed in previous reports of overgeneralization in clinical populations compared to healthy controls for behavioral responses (e.g., Lissek et al., 2010, 2014a). Our overgeneralization hypothesis in individuals with elevated Misophonia was also driven by the large overlap in symptomology between anxiety and fear-related disorders and Misophonia (Ginsburg et al., 2006; Hadjipavlou et al., 2008; Edelstein et al., 2013; Wu et al., 2014; Dozier, 2015; Webber and Storch, 2015; Zhou et al., 2017; Quek et al., 2018; Erfanian et al., 2019; Potgieter et al., 2019; Jager et al., 2020; McKay and Acevedo, 2020; Guetta et al., 2022), suggesting that individuals with Misophonia may display similar overgeneralization learning. Furthermore, several (Dozier, 2015; Kumar et al., 2021) have proposed that emotional responses to auditory “trigger” cues may over time generalize to other stimuli via associative learning processes.

We also considered the alternative hypothesis that (H3B) a sharpened tuning response pattern (Figure 1B) across our dependent variables would be found in individuals with greater MSS scores, as seen in previous work assessing sensory responses in socially anxious individuals (Stegmann et al., 2020). Such a response pattern would indicate suppression of the most similar GS, resulting in sharpening in sensory systems (McTeague et al., 2015). We included an all-or-nothing discrimination learning model (Figure 1C) to assess the possibility that (H3C) the CS+ alone would elicit heightened responses in our dependent variables, with little to no difference in response to the other GS, an effect observed previously for alpha power changes in visual aversive conditioning paradigms (Friedl and Keil, 2020, 2021).

Finally, we predicted that individuals endorsing greater Misophonia symptomology would also exhibit larger response change scores for the CS+ from the habituation to acquisition phases compared to those with less Misophonia symptomology. This was assessed by correlating individuals’ MSS scores with calculated change scores for self-reported behavioral ratings of valence and arousal, and parietal alpha-band power. If supported, these findings would suggest that individuals endorsing Misophonia are more likely to have adverse and arousing reactions, as well as greater attentional processing, to auditory stimuli that have acquired adverse attributes.

## Materials and Methods

The study design and hypotheses are part of a larger project that was preregistered prior to data collection^1^. Here, we report initial preliminary findings for the aversive generalization task, and our planned analyses pertaining to alpha-band power. In addition, we have included results from an assessment of loudness discomfort level thresholds, aimed to capture one facet of hyperacusis, a disorder of broad hypersensitivity to the volume of auditory stimuli. These measurements were included to examine the extent to which relations observed between self-reported Misophonic symptoms and the dependent variables were specific to Misophonia symptoms or partly explained by loudness discomfort as is characteristic for hyperacusis.

### Participants

This report represents a preliminary analysis of a subset of data from an ongoing study. For the data discussed in the present article, 36 participants were recruited through online advertisements, flyers, and existing data bases. Participants were recruited and prescreened to include individuals scoring high on the Misophonia Symptom Scale (MSS), detailed below. They were either paid 20 USD per hour or received class credit. All participants provided informed consent prior to participation in accordance with the Declaration of Helsinki, with all procedures approved by the institutional review board at the University of Florida. Participants were at least 18 years of age, reported normal or corrected-to-normal vision, and indicated no history of seizures. Two participants were excluded from data analysis due to having over 50% of EEG trials containing artifacts (*n* = 1), and technical errors (i.e., program crash) during data collection (*n* = 1). This resulted in a total of 34 (21 Female; *M*_*age*_ = 19.85, *SE*_*age*_ = 0.29) participants used for data analyses (see Table 1 for full demographics).

**TABLE 1:** Demographic information.

Variable	*N* (%)	*M*_*age*_	*SE*_*age*_
**Sex**			
Male	13 (37.14%)	20.08	0.59
Female	21 (60.00%)	19.71	0.30
**Gender**			
Man	13 (37.14%)	20.08	0.59
Woman	20 (57.14%)	19.70	0.32
Non-binary	1 (2.86%)	20.00	N/A
**Ethnicity**			
Hispanic	28 (80.00%)	19.79	0.33
Non-hispanic	6 (17.14%)	20.17	0.60
**Race**			
Asian	4 (11.43%)	21.25	1.49
Black	1 (2.86%)	21.00	N/A
White	29 (82.865)	19.62	0.27

*Demographics are provided for the entire sample used for data analyses. N/A provided for SE_age_ due to no variability in the respective demographic categories.*

Participants completed the Misophonia Questionnaire (Wu et al., 2014) and a set of additional questionnaires capturing symptoms in the OCD, Fear, Anxiety, and Depression spectrum. Only data from the Misophonia Questionnaire are included in the present report.

### Materials and Procedure

#### Misophonia Measures

Symptoms of Misophonia were quantified using the Misophonia Symptom Scale (MSS), a sub-scale of the Misophonia Questionnaire (Wu et al., 2014; Supplementary Appendix Table 1). This seven-question measure assesses the degree to which individuals experience sound sensitivities to specific circumstances, such as people making throat or nasal sounds. Specifically, this questionnaire requires participants to rate how bothered they feel when hearing these specific sounds on a 5-point Likert scale ranging from 0 (i.e., Never) to 4 (i.e., Always), yielding a potential sum score between 0 and 28. The MSS has demonstrated high internal consistency (i.e., α = 0.83–0.86; Wu et al., 2014; McErlean and Banissy, 2018), with our sample showing similar internal consistency (α = 0.85). Wu et al. (2014) considered scores 14 or greater on the MSS as reflective of elevated Misophonia symptomology. In this report, we use the MSS as a continuous variable, with MSS scores in our sample ranging from 0 to 20 (*M* = 9.55, *SE* = 0.88).

#### Self-Assessment Manikin Measures

**FIGURE 2:**
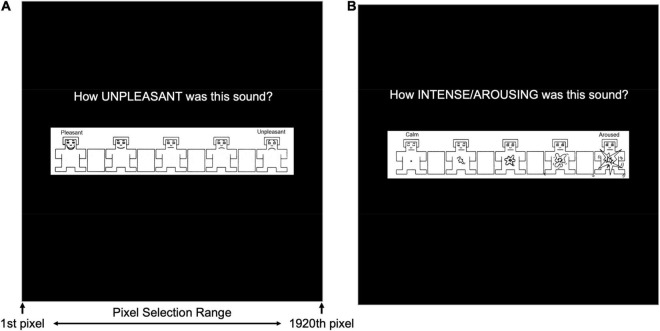
Self-Assessment Manikin (SAM) presented to during the early and late stages of both the habituation and acquisition phases in the auditory aversive generalization task. **(A)** Measures of valence quantified by pixel number (i.e., 1–1,920) corresponding with participants’ mouse click on an *x*-axis of the monitor’s length. **(B)** Measures of arousal were similarly represented by pixel number corresponding with participants’ mouse clicks.

Self-Assessment Manikin (SAM; Bradley and Lang, 1994) ratings were collected for valence (Figure 2A) and arousal (Figure 2B) during early and late periods of both the habituation and acquisition phases of the aversive generalization task. SAM ratings were assessed following a presentation of each pitch during each assessment period (i.e., early/late habituation, and early/late acquisition). Five manikins were presented for valence and arousal, and participants were required to click on a continuous scale to rate how pleasant/unpleasant (i.e., valence) and calm/aroused (i.e., arousal) they felt after hearing each tone. All responses were recorded as pixel location (i.e., *x*-axis, ranging from 1 to 1,920 pixels) where participants clicked to indicate their valence or arousal.

#### Loudness Discomfort Level Testing

Loudness discomfort levels (LDLs), a characteristic of hyperacusis, were assessed by presenting individual sine-wave tones varying in amplitude for one second at one of five randomly presented pitches (i.e., 320, 544, 925, 1,572, and 2,673 Hz). Next, participants were provided a dichotomous choice to increase the loudness or not. If they selected “yes” to increase the loudness, the tone at that respective pitch was presented in the subsequent trial at an increased loudness level. If the participant selected “no” to increase the loudness, the next randomly selected pitch would be presented at the minimal loudness level. Loudness levels, measured with an audiometer, ranged in steps of 1–10 (ranging from ∼69 to ∼91 dBA), increasing approximately 2.5 dB for every unit increase in loudness. If participants reached the max loudness level for a given pitch (i.e., loudness level of 10), they would be presented with the next pitch regardless of their choice. Measures of hyperacusis sensitivity were calculated as the sum of loudness levels across each pitch, ranging from 1 to 50, with higher values indicating higher auditory tolerance thresholds. It is important to note that LDL measures do not serve as a complete assessment of hyperacusis. The assessment was presented using Psychtoolbox code (Brainard, 1997) on a Cambridge research systems Display ++ monitor (1,920 × 1,080, 120 Hz refresh rate) at 120 cm distance from the participant, and auditory stimuli were presented through two Behringer Studio 50 speakers arranged symmetrically behind the participant at ear level, at a 30 cm distance.

#### Auditory Stimuli

A sine-wave tone was presented for 4 s (88,001 sample points) at three different pitches, consisting of frequencies of 320, 541, and 914 Hz chosen from an exponential pitch function. We chose three pitches based on the results of extensive pilot work prior to the start data acquisition in the current study. In this pilot work, we tested various ranges of pitches, with up to 5–7 pitch conditions being presented spaced between 320 and 914 Hz. Using these larger condition designs, we found that participants failed to identify the CS+, indicating failure to learn contingencies between a specific pitch and US. However, when we tested a design using only these three pitches, participants correctly learned which pitch predicted the CS+. Thus, although these frequencies may be in the pleasantness range (Patchett, 1979), as discussed in our results below, we found significant differences in behavioral measures of valence and arousal for these stimuli following conditioning.

A cosine-wave was generated to create onset and offset-ramps for each pitch. The loudness of each pitch was normalized by dividing each pitch’s amplitude at a given sample point by its respective frequency. This resulted in normalizing loudness levels to 70 dBA to ensure consistent loudness levels were presented for each frequency. In addition, a 91 dBA white noise was also generated, using white noise with 22,001 sample points, multiplied with a ramp-off, ramp-down cosine square window of 5 sample points to avoid popping at the beginning and end. This loud white noise stimulus served as the US, and was presented during the final second of the 4 s tone presentation of the sinewave tone designated the CS+. The duration of 1 s was chosen because previous work has shown that loud noise USs are most effective when longer than 500 ms (Sperl et al., 2016). In addition, the final second was chosen because Pavlovian conditioning is most effective when the CS+ and US co-terminate after having overlapped for a period of time (Kamin, 1956). This white noise was paired with the 320 Hz tone, with both the tone and white noise co-terminating. Thus, the 320 Hz pitch served as the CS+ (100% reinforcement rate), while the 541 and 914 Hz pitches were never paired with the white noise, allowing for generalization learning to occur across a gradient of pitches, (541 Hz serving as the GS1 and 914 Hz as the GS2). All tones were multiplied by a 41.2 Hz cosine envelope for a separate set of analyses not reported here (see our preregistration for more details). All auditory stimuli were presented through two Behringer Studio 50 speakers.

#### Auditory Aversive Generalization Task

**FIGURE 3:**
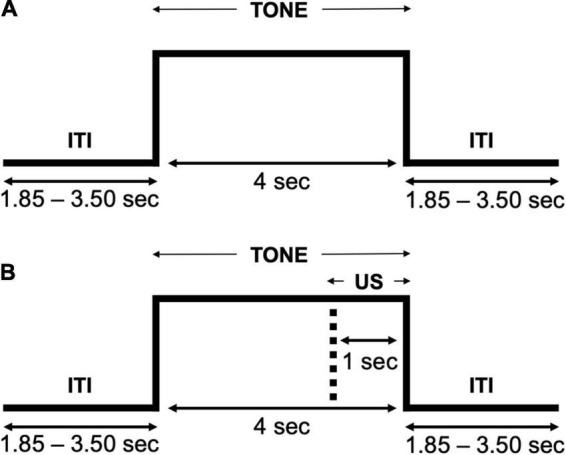
Trial flow of the auditory aversive generalization task. **(A)** The habituation phase presented a tone (each pitch) without any white noise US pairings. **(B)** The acquisition phase presented a tone (each pitch) with the lowest pitch being paired with the white noise US.

Participants completed an aversive generalization task consisting of tones presented at three different pitches (i.e., CS+, GS1, and GS2) over a habituation and acquisition phase. Given that the task primarily required active listening, no practice trials were presented to participants. However, all participants were informed that they would be required to rate the sounds presented using a mouse to click a location on a scale presented several times throughout the experiment (i.e., SAM ratings). No white noise presentations occurred during the habituation phase (Figure 3A), and only the tone serving as a CS+ (i.e., 320 Hz) was paired with this US during the acquisition phase (Figure 3B). Participants completed a total of 240 trials (80 per condition), 120 in the habituation phase (40 per condition), and 120 in the acquisition phase. SAM ratings for each tone were acquired following trials 10 and 90 in each of the two phases, allowing for early and late behavioral assessments in both habituation and acquisition phases. The first and third trials in the acquisition phase were designed to be CS+, serving as booster trials to facilitate learning, and the remaining conditions were randomized, with the constraint that not more than 2 CS+ trials would occur in sequence. Each trial began with a central white fixation dot (0.8° of visual angle) presented throughout the entire task, excluding when SAM ratings were presented. Following a variable inter-trial interval (ITI; 1.85–3.50 s), a tone at a specific pitch was presented for 4 s. All stimuli were presented using Psychtoolbox code (Brainard, 1997) on a Cambridge research systems Display ++ monitor (1,920 × 1,080, 120 Hz refresh rate) at 120 cm distance from the participant, and auditory stimuli were presented through two Behringer Studio 50 speakers arranged symmetrically behind the participant at ear level, at a 30 cm distance. The entire experiment (i.e., completion of the task, survey measures, and EEG application) took approximately an hour and 15 min per participant.

### Data Acquisition and Signal Processing

#### Differential Aversive Conditioning

Continuous EEG data were recorded using an Electrical Geodesics (EGI) high-impedance system with a 128-channel (Ag-AgCl electrodes) HydroCel net. Online data were recorded at a 500 Hz sampling rate, referenced to the vertex sensor (Cz), with impedances kept below 60 kΩ. Online Peyk et al. (2011) Butterworth low-pass (3 dB point at 60 Hz) and high-pass (3 dB point at 0.1 Hz) filters were applied throughout recording. Data were then re-filtered offline using Butterworth low-pass (10th order, 3 dB point at 30 Hz) and high-pass (3rd order, 3 dB point at 1 Hz) filters, and were re-referenced to the average reference (i.e., averaged across all sensors). EEG data were segmented into epochs of 3.6 s (1,801 sample points), 600 ms (300 sample points) prior to the onset of the tone and 3,000 ms (1,501 sample points) following the tone onset. This 3,000 ms duration was selected as opposed to the 4,000 ms presentation time to prevent any artifactual confounds resulting from the presentation of the US within the final 1,000 ms. Epoched trials then underwent artifact rejection based on the Statistical Correction of Artifacts in Dense Array Studies (SCADS) procedure (Junghöfer et al., 2000), in which data quality indices (absolute value, standard deviation, and maximum of differences across time points) for each channel and trial were calculated. Eye movements were corrected with regression-based EOG correction methods (Schlögl et al., 2007, 2009) using HEOG and VEOG sensors. Participants with trials containing excessive artifacts (i.e., >50% of all trials rejected) were removed from analyses. This procedure resulted in an average 9.2 trials (*SE* = 1.30) of the total 40 trials per condition being rejected in the remaining participants used for analyses. Importantly, the total number of trials retained did not significantly differ between conditions within phases (habituation: CS+ = 32.0; GS1 = 33.7; GS2 = 33.9; acquisition: CS+ = 28.4; GS1 = 28.4; GS2 = 27.71), but differed between the habituation and acquisition phases, *t*(33) = 4.21, *p* ≤ 0.001, BF_10_ = 141.4.

#### Alpha-Band Power Quantification

Artifact-free single trial data were transformed into the time-frequency domain by convolving the EEG data with a family of complex Morlet wavelets with center frequencies (*f*) between 2.50 and 27.49 Hz, in steps of 0.2776 Hz. A Morlet constant (i.e., *m*) was calculated by dividing the center frequencies by the frequency smoothing value (i.e., sigma_f), using the formula: *m* = *f/*sigma_f = 10. This Morlet constant was chosen to optimize the trade-off between temporal smoothing (sigma_t) and frequency smoothing for the lower alpha-band frequencies targeted by the present research [i.e., sigma_f = 1/(2*pi*sigma_t)]. We obtained a sigma_f = 0.86 Hz and a sigma_t = 185 ms at our lowest center frequency of interest (i.e., 8.61 Hz). The absolute value of the convolution between that data and the complexed wavelets was obtained, and served as our estimate of time-varying power (Tallon-Baudry and Bertrand, 1999).

Next, all trials were averaged by condition, and total power was baseline corrected as the percentage change from a 222 ms interval preceding the tone onset (−422 to −202 ms prior to tone onset), to accommodate edge artifacts of the wavelet transform and account for temporal smoothing factors. We used baseline division given that alpha-band power was present in the baseline period, and the amount of reduction in percent has been shown to co-vary meaningfully with a range of experimental tasks (Jensen and Mazaheri, 2010; Foxe and Snyder, 2011). Alpha-band power was measured by averaging the time-varying power across wavelets ranging from 8.60 to 11.13 Hz.

### Statistical Analyses

#### Overview

The dependent variables consisted of behavioral ratings of valence and arousal for each tone across habituation and acquisition phases of the aversive generalization task, and parietal alpha power. Auditory tolerance thresholds (i.e., hyperacusis LDL test) were included as covariates in our hierarchal linear model analyses of behavioral data. All frequentist analyses (e.g., repeated measures ANOVAs) included Greenhouse–Geisser adjustments when sphericity assumptions were violated. Significant main effects and interactions were decomposed using Bonferroni corrected comparisons. We also conducted Bayes Factor analyses to assess the degree of evidence supporting the null versus alterative hypothesis (Dienes, 2014, 2016; Jarosz and Wiley, 2014; Lee and Wagenmakers (2014); Wagenmakers et al., 2016, 2018a,2018b; Keysers et al., 2020; Lakens et al., 2020; van Doorn et al., 2021). Bayes Factor 10 (BF_10_) values are represented on a continuous scale, as opposed to the dichotomous scale affiliated with frequentist approaches (e.g., *p*-values). Although there is debate in terms of interpretation criteria for BF_10_ outcomes (see Jeffreys, 1939; Kass and Raftery, 1995; Jarosz and Wiley, 2014; van Doorn et al., 2021), many agree that BF_10_ scores near 0 provide strong support for the null hypothesis, with the strength of this evidence decreasing as the BF_10_ becomes larger, and thus evidence for the alternative hypothesis becoming strengthened. We chose multivariate Cauchy priors (fixed effects = 0.5, covariates = 0.354) given the possibility for any statistical test outcome being possible, resulting in a uniform prior distribution (Lee and Vanpaemel, 2018; van Doorn et al., 2021).

#### Behavioral Valence and Arousal

To assess how MSS scores influenced raw valence and arousal behavioral ratings, including their change from habituation to acquisition phases, we conducted hierarchal linear model analyses using maximum likelihood (ML) methods. First, we conducted a series of step-wise model testing, in which we began with an intercept-only model, with intercepts allowed to vary randomly by participant (level 3), and added a predictor variable in each model iteration, until the addition of predictor terms no longer significantly contributed explaining the variability of our valence/arousal measures. Specifically, we assessed the following variables in each of the respective iterations: (1) Phase (level 1), (2) Pitch (level 2), (3) MSS score (level 3), (4) Pitch × Phase (cross-level) and MSS score, (5) Pitch × Phase × MSS score (cross-level). Auditory tolerance threshold scores (level 3), measured using LDLs, and MSS scores were mean-centered, with the auditory tolerance threshold scores serving as covariates in all models. Our Pitch factor consisted of the CS+, GS1, and GS2 conditions (3 levels of the factor), and the Phase factor included early/late habituation and early/late acquisition (4 levels of the factor). Only fixed effects were assessed. Our model comparisons yielded a final model including the predictor variables of Pitch, Phase, Pitch × Phase, MSS score, and the covariate of auditory tolerance threshold (see Table 2 for model comparison breakdown).

**TABLE 2:** Raw behavioral data comparison outcomes.

	Valence			Arousal		
Contrast	χ^2^ value	df	*p*-value	χ^2^ value	df	*p*-value
Rating = Intercept – Rating = Phase + Intercept	32.25	4	<0.001***	63.26	4	<0.001***
Rating = Phase + Intercept – Rating = Phase + Pitch + Intercept	39.22	2	<0.001***	73.38	2	<0.001***
Rating = Phase + Pitch + Intercept – Rating = Phase + Pitch + MSS + Intercept	6.70	1	0.010*	10.84	1	<0.001***
Rating = Phase + Pitch + MSS + Intercept – Rating = Phase × Pitch + MSS + Intercept	102.47	6	<0.001***	147.49	6	<0.001***
Rating = Phase × Pitch + MSS + Intercept – Rating = Phase × Pitch × MSS + Intercept	8.60	11	0.658	1.59	3	0.662

All models included auditory tolerance threshold scores as covariates. Bold p-values indicate significant model comparisons. *p < 0.05, ***p < 0.001.

Next, we assessed the degree to which the valence and arousal ratings across CS+ and GS conditions fit one of the three learning models discussed above (i.e., generalization, sharpening, and all-or-nothing) within each behavioral assessment phase (i.e., early/late habituation and acquisition). This was done by computing a series of weights for each pitch (i.e., CS+, GS1, and GS2) based on our hypothesized gradient response pattern, with the sum of these weights equal to zero in each model (Figure 1). Specifically, the following weights were applied to each pitch in the respective model: generalization ∼ CS+ = 1, GS1 = 0.75, GS2 = −1.75; sharpening ∼CS+ = 1, GS1 = −1.5, GS2 = 0.5; and all-or-nothing ∼ CS+ = 2, GS1 = −1, GS2 = −1. These weights were multiplied for each pitch’s valence and arousal score, separately, within each phase. This resulted in a single value reflecting the relative strength of each model’s fit within each assessment phase. Importantly, greater values reflected stronger model fits for the behavioral data. Following the logic of our hierarchal linear model analyses for raw valence and arousal ratings, we conducted another series of step-wise model comparisons, including the same predictor variables in the order tested previously. However, we replaced the Pitch factor with a Model factor (i.e., generalization, sharpening, and all-or-nothing) to test the degree to which each model represented these behavioral data over each phase. These step-wise model comparisons resulted in a final model with the predictor variables of Pitch, Model, Pitch × Model, and the covariate of auditory tolerance threshold score (see Table 3). Critically, MSS score was not a significant contributor to predicting variability in model strength in valence or arousal, and was thus excluded in our final model.

**TABLE 3:** Modeled behavioral data comparison outcomes.

	Valence			Arousal		
Contrast	χ^2^ value	df	*p*-value	χ^2^ value	df	*p*-value
Rating = Intercept – Rating = Phase + Intercept	195.67	4	<0.001***	235.06	4	<0.001***
Rating = Phase + Intercept – Rating = Phase + Model + Intercept	17.30	2	<0.001***	31.90	2	<0.001***
Rating = Phase + Model + Intercept – Rating = Phase + Model + MSS + Intercept	0.87	1	0.351	0.02	1	0.894
Rating = Phase + Model + Intercept – Rating = Phase × Model + Intercept	35.77	6	<0.001***	51.94	6	<0.001***
Rating = Phase × Model + Intercept – Rating = Phase × Model × MSS + Intercept	6.76	12	0.873	8.46	12	0.748

All models included auditory tolerance threshold scores as covariates. Bold p-values indicate significant model comparisons. ***p < 0.001.

#### Alpha-Band Power

Because of the higher dimensionality of EEG data (e.g., time and sensors in addition to conditions), we used a different approach for analyzing alpha-band power than what was done for self-reported valence and arousal. Two approaches were then taken for alpha-band power statistical analyses. First, time-varying alpha-band power (% change from baseline) was extracted in two separate time windows, one early (i.e., 300–800 ms post-tone onset) and one late (800–1200 ms post-tone onset), and averaged across a parietal sensor cluster containing the central parieto-occipital sensor POz and its 5 nearest neighboring sensors. The second approach used all sensors and time points, controlled by a mass-univariate permutation approach (Blair and Karniski, 1993), described in more detail below.

A 2 (Phase: habituation and acquisition) × 3 (Pitch: CS+, GS1, and GS2) mixed ANOVA was conducted for parietal alpha-band power to test the prediction that the CS+ in the acquisition phase would selectively elicit the largest response compared to all other conditions, including the CS+ in the habituation phase. Next, we computed a change score for each pitch (i.e., CS+, GS1, and GS2), from the habituation to acquisition phase. This was done by subtracting the raw alpha-band power value in a pitch condition’s acquisition phase from the alpha-band power in that same pitch condition’s habituation phase (i.e., acquisition – habituation = change score or _Δ_). This resulted in change scores of CS+_Δ_, GS1_Δ_, and GS2_Δ_. Similar to our behavioral analyses, we fit these change scores with our learning models (i.e., generalization, sharpening, and all-or-nothing), resulting in the final learning models: generalization ∼CS+_Δ_ = 1, GS1_Δ_ = 0.75, GS2_Δ_ = −1.75; sharpening ∼ CS+_Δ_ = 1, GS1_Δ_ = −1.5, GS2_Δ_ = 0.5; and all-or-nothing ∼ CS+_Δ_ = 2, GS1_Δ_ = −1, GS2_Δ_ = −1. This was done for both alpha-band intervals (i.e., early and late).

After computing weighted alpha-band power change scores, we conducted *F* contrasts on the two selected time ranges (i.e., early and late) and the parieto-occipital electrode clusters to examine how similar the raw change scores for each pitch were to the predicted model trend (i.e., generalization, sharpening, and all-or-nothing). The same *F*-contrasts were also separately computed for each sensor and time point in the alpha-band power change score time series for the three pitches, resulting in a mass-univariate spatiotemporal map of *F*-values. These maps were controlled by a permutation technique (Blair and Karniski, 1993; McTeague et al., 2015), further described below in our correlational analyses with MSS scores, resulting in a permutation controlled threshold of F*_*crit*_* = 7.88. These maps served as manipulation and data quality checks, and were expected to indicate which learning model was most strongly fit our alpha-band power changes. To quantify the linear relationship between learning-induced alpha-band power changes and MSS score as a continuous variable, we quantified each variable’s fit with the three competing learning models for each participant, computing the inner product between the resulting three values per dependent variable (i.e., early alpha-band time window, late alpha-band time window, and mass univariate approach for alpha-band power), and the model weights for each learning model. These values represented a direct measure of the strength of learning-induced changes, and were then correlated with MSS using Pearson’s *r* correlations, with and without controlling for auditory tolerance threshold score.

For the mass univariate evaluation of correlations between learning model fits of alpha-band power change scores and MSS scores, we obtained Pearson’s *r*-values (corresponding to a significance level of 0.05) by calculating distributions of *r*-values on data shuffled between the conditions and within each participant (i.e., 1,000 permutations). Specifically, we randomly permuted the three change scores obtained for each pitch by subtracting alpha-band power in acquisition from habituation, randomly within each participant 1,000 times, and then computed *F*-values for each sensor and time point (Blair and Karniski, 1993). The same approach was taken when correlating learning model fits of alpha-band power change scores with MSS scores. Next, the minimum and maximum and of each Pearson’s *r* distribution was determined and stored in an *r*_*min*_ and *r*_*max*_ distribution, respectively, with each index having 1,000 values corresponding with the 1,000 permutations. The 2.5th and 97.5th percentiles from these *r*_*min*_ and *r*_*max*_ distributions were used as critical values. For the present data, these mass univariate correlation thresholds were −0.51 and +0.50. Only empirical correlations crossing this defined threshold were considered statistically significant.

## Results

### Behavioral Outcomes

#### Raw Valence and Arousal

**FIGURE 4:**
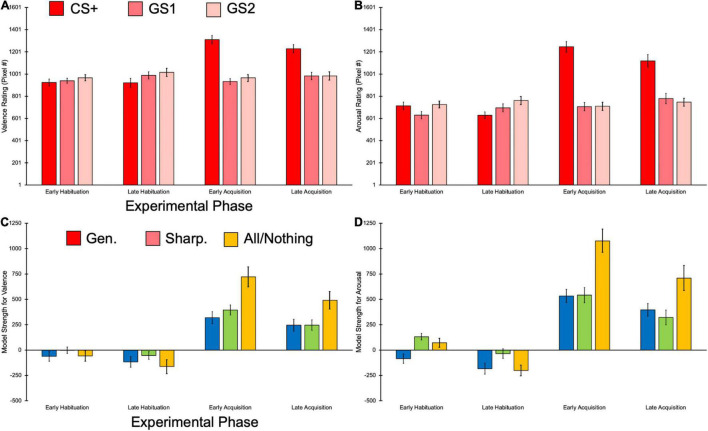
Bar plots showing the raw and model strength scores for self-reported behavioral data. **(A)** The CS+ elicited more negative ratings than the other pitches, and this was driven by the acquisition phases. **(B)** The CS+ also was rated as more arousing than the other GSs, once more being primarily observed in the acquisition phases. **(C)** The All-or-Nothing model provided the best fit for valence rating data, an effect driven by the acquisition phases. **(D)** Arousal ratings were also better fit with the All-or-Nothing learning model, which was primarily found in the acquisition phases. Error bars represent ±1 standard error.

**FIGURE 5:**
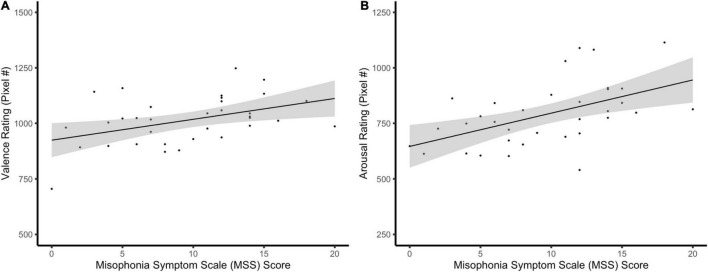
Scatter plots showing the association between self-reported behavioral variables and MSS scores. **(A)** Valence ratings, regardless of pitch or phase, were positively associated with MSS scores, even after controlling for auditory tolerance threshold scores. **(B)** MSS scores were positively related to arousal ratings across pitch and phase after controlling for auditory tolerance threshold scores.

In the hierarchal linear model predicting raw valence scores from the predictor variables of Pitch, Phase, Pitch × Phase, MSS, and the covariate of auditory tolerance threshold, we observed a main effect of Pitch, *F*(2,374) = 27.19, *p* < 0.001, $\eta_{\text{p}}^{2}$ = 0.13, BF_10_ = 32.61e + 5. The CS+ elicited significantly more negative valence ratings than GS1 [*t*(385) = 6.77, *p* < 0.001] and GS2 [*t*(385) = 5.67, *p* < 0.001], but GS1 did not significantly differ in valence than GS2, *t*(385) = −1.11, *p* = 0.808. A main effect of Phase was also found [*F*(3,374) = 15.68, *p* < 0.001, $\eta_{\text{p}}^{2}$ = 0.11, BF_10_ = 10.87e + 2], with significantly more negative valence ratings being reported in early acquisition compared to early [*t*(385) = 5.46, *p* < 0.001] and late habituation, *t*(385) = 4.11, *p* < 0.001. Valence was also rated as more negative in late acquisition compared to early [*t*(385) = 5.25, *p* < 0.001] and late habituation, *t*(385) = 3.90, *p* < 0.001. No significant differences in valence between early and late habituation [*t*(385) = −1.35, *p* > 0.999], and early and late acquisition were observed, *t*(385) = 0.21, *p* > 0.999. In addition, we found a Pitch × Phase interaction [*F*(6,374) = 19.65, *p* < 0.001, $\eta_{\text{p}}^{2}$ = 0.24, BF_10_ = 45.91e + 18], demonstrating that the CS+ during the acquisition phases was rated as more negative compared to the other GS and the CS+ in the habituation phases (Figure 4A). *Post hoc* Bonferroni comparisons for this interaction are reported in Supplementary Appendix Table 2. Importantly, we observed a main effect of MSS [*F*(1,34) = 7.41, *p* = 0.010, $\eta_{\text{p}}^{2}$ = 0.18, BF_10_ = 620.78], such that valence ratings were predicted to be approximately 9.60 (95% CI [2.35, 15.78] pixels further to the right (i.e., more negative) for every unit increase in MSS (Figure 5A). Auditory tolerance threshold scores were non-significant in this model, *F*(1,34) = 1.07, *p* = 0.307, $\eta_{\text{p}}^{2}$ = 0.03, BF_10_ = 0.65.

Our model predicting arousal ratings yielded a main effect of Pitch [*F*(2,374) = 60.14, *p* < 0.001, $\eta_{\text{p}}^{2}$ = 0.24, BF_10_ = 55.16e + 7], with the CS+ being reported as having greater arousal than the GS1 [*t*(385 = 10.01, *p* < 0.001] and GS2, *t*(385) = 8.53, *p* < 0.001. Arousal ratings did not significantly differ between the GS1 and GS2, *t*(385) = −1.48, *p* = 0.416. We also observed a main effect of Phase [*F*(3,374) = 38.05, *p* < 0.001, $\eta_{\text{p}}^{2}$ = 0.23, BF_10_ = 10.87e + 6], with participants reporting significantly higher arousal ratings in early acquisition compared to early [*t*(385) = 7.65, *p* < 0.001] and late habituation, *t*(385) = 7.44, *p* < 0.001. Significantly greater arousal ratings were also reported in the late acquisition relative to the early [*t*(385) = 7.45, *p* < 0.001] and late habituation phases, *t*(385) = 7.23, *p* < 0.001. No significant differences in arousal were found between early and late habituation [*t*(385) = −0.21, *p* > 0.999] and early and late acquisition phases, *t*(385) = 0.20, *p* > 0.999. Similar to our valence findings, we observed a significant Pitch × Phase interaction [*F*(6,374) = 30.13, *p* < 0.001, $\eta_{\text{p}}^{2}$ = 0.33, BF_10_ = 12.39e + 33], suggesting that participants rated the CS+ during the acquisition phases as being more arousing than the other GS and the CS+ in the habituation phases (Figure 4B). All *post hoc* follow-up comparisons for this interaction can be seen in Supplementary Appendix Table 2. MSS also had a main effect [*F*(1,34) = 12.77, *p* = 0.001, $\eta_{\text{p}}^{2}$ = 0.27, BF_10_ = 57.98e + 3], such that arousal ratings were approximately 14.09 (95% CI [6.14, 22.04] pixels further to the right (i.e., more arousing) for every unit increase in MSS (Figure 5B). Unlike results for valence, auditory tolerance threshold scores significantly predicted arousal ratings, *F*(1,34) = 5.40, *p* = 0.026, $\eta_{\text{p}}^{2}$ = 0.14, BF_10_ = 63.05. Specifically, arousal ratings were predicted to be 3.00 (95% CI [0.40, 5.61] pixels further to the right (i.e., more arousing) for every unit increase in auditory tolerance threshold score.

In summary, overall valence measures, regardless of pitch and experimental phase, were associated with MSS scores, but not with auditory tolerance threshold scores. In contrast, arousal ratings were associated with both MSS and auditory tolerance threshold scores, regardless of pitch and experimental phase. Nonetheless, the CS+ demonstrated more negative valence and greater arousal than the GS1 and GS2, which did not significantly differ. However, this effect only was found during the acquisition, as expected.

#### Learning Model Comparisons

The fit of generalization, sharpening, and all-or-nothing learning models to the rating data was examined next. The hierarchal linear model predicting model fit strength for valence from Pitch, Model, Pitch × Model, and the covariate of auditory tolerance threshold yielded a main effect of Model, *F*(2,374) = 9.74, *p* < 0.001, $\eta_{\text{p}}^{2}$ = 0.05, BF_10_ = 1.45. Bonferroni *post hoc* comparisons indicated that the all-or-nothing model yielded significantly greater strength, or fit with the valence data, compared to the generalization [*t*(385) = −4.27, *p* < 0.001] and sharpening models, *t*(385) = −2.87, *p* = 0.013. The generalization and sharpening models did not significantly differ in model strength, *t*(385) = −1.40, *p* = 0.49. We also observed a main effect of phase [*F*(3,374) = 98.76, *p* < 0.001, $\eta_{\text{p}}^{2}$ = 0.44, BF_10_ = 63.94e + 28], with late acquisition phase showing greater model strength compared to the early habituation [*t*(385) = −8.95, *p* < 0.001] and late habituation phases [*t*(385) = −10.65, *p* < 0.001], but weaker model strength than the early acquisition phase, *t*(385) = 3.69, *p* = 0.002. The early acquisition phase also held significantly greater model strength than the early habituation [*t*(385) = −12.64, *p* < 0.001] and late habituation phases [*t*(385) = −14.34, *p* < 0.001], and no significant differences in model strength were found between the early and late habituation phases, *t*(385) = 1.70, *p* = 0.541. A Model × Phase interaction was found [*F*(6,374) = 6.26, *p* < 0.001, $\eta_{\text{p}}^{2}$ = 0.09, BF_10_ = 31.71e + 31], suggesting that the all-or-nothing model held the best fit for valence data, but primarily in the acquisition phases (Figure 4C). All *post hoc* comparisons for this interaction can be seen in Supplementary Appendix Table 3. Auditory tolerance threshold scores did not significantly predict model strength, *F*(1,34) < 0.01, *p* = 0.967, $\eta_{\text{p}}^{2}$ < 0.01, BF_10_ < 0.01.

We found a main effect of Model [*F*(2,374) = 19.13, *p* < 0.001, $\eta_{\text{p}}^{2}$ = 0.09, BF_10_ = 24.78] in our analysis predicting model strength for arousal ratings, such that the all-or-nothing model was a significantly better fit for arousal data than the generalization [*t*(385) = −5.94, *p* < 0.001] and sharpening models [*t*(385) = −4.12, *p* < 0.001], with these latter models showing non-significant differences in model strength, *t*(385) = −1.78, *p* = 0.226. A main effect of Phase was also seen [*F*(3,374) = 136.02, *p* < 0.001, $\eta_{\text{p}}^{2}$ = 0.52, BF_10_ = 48.56e + 36], with early acquisition showing greater model fit than late acquisition [*t*(385) = 4.99, *p* < 0.001], early habituation [*t*(385) = −14.01, *p* < 0.001], and late habituation phases, *t*(385) = −17.72, *p* < 0.001. Model strength in the late acquisition phase was also a better fit for arousal data compared to model strength in the early habituation [*t*(385) = −9.02, *p* < 0.001] and late habituation phases [*t*(385) = −12.73, *p* < 0.001], and the early habituation phase arousal data had greater model strength than the late habituation phase, *t*(385) = 3.71, *p* = 0.001. Similar to our valence results, we also obtained a Model × Phase interaction [*F*(6,374) = 9.29, *p* < 0.001, $\eta_{\text{p}}^{2}$ = 0.13, BF_10_ = 48.39e + 43], with the main findings of our Bonferroni *post hoc* comparisons (Supplementary Appendix Table 3) demonstrating the all-or-nothing model held the best fit for the arousal data, but this was only the case in the acquisition phases (Figure 4D). Auditory tolerance threshold scores were non-significant in predicting model strength, *F*(1,34) = 0.57, *p* = 0.455, $\eta_{\text{p}}^{2}$ = 0.02, BF_10_ = 0.206.

Taken together, these outcomes suggests that neither MSS nor auditory tolerance threshold scores account for the variability in predicting valence ratings based on different learning models. Similar model strength outcomes were also observed for arousal ratings, with neither of these predictor variables significantly contributing to model strength. Despite these outcomes, our behavioral data suggests the all-or-nothing model was a better fit for both valence and arousal data compared to the generalization and sharpening models, but only during acquisition.

### Alpha-Band Power Outcomes

**FIGURE 6:**
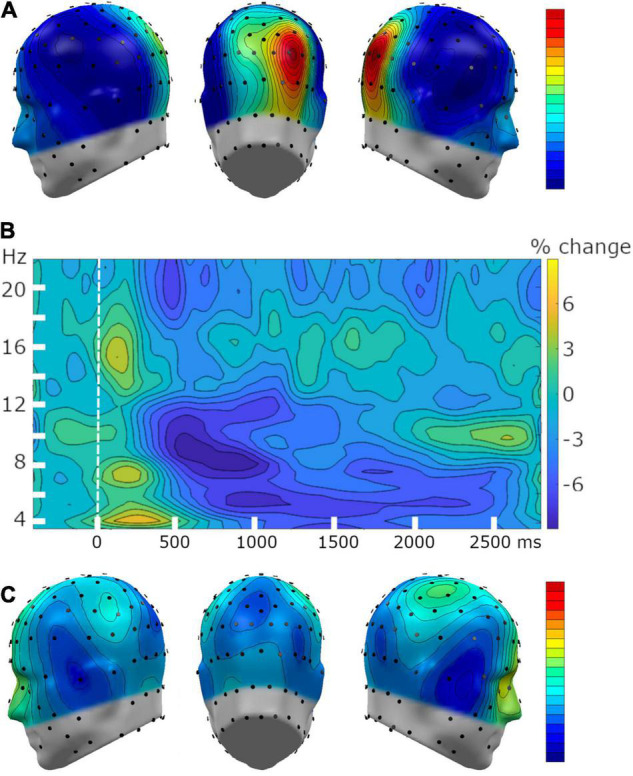
Grand mean time-frequency changes, with a focus on power reduction in the alpha-band (8–12 Hz). **(A)** Topography of the grand mean (*n* = 34) alpha power during the pre-tone baseline segment demonstrated a typical parietal alpha-band power topography. **(B)** Grand mean time-frequency representation of baseline-adjusted power changes at sensor POz and its 5 nearest neighboring sensors. **(C)** The topography of the alpha-band power reduction relative to baseline, averaged across a time range from 300 to 1,200 ms following the onset of the pitch.

Parietal alpha-band power was present throughout the baseline segment, and showed the expected parietal topographical distribution (Figure 6A). The tone onset prompted decrease in parietal alpha-band power, which spanned a frequency range from 8 to 12 Hz across a time window between 300 to 1,200 ms post-pitch. As described above, alpha-band power averaged separately across time points into two adjacent analytical windows (i.e., 300–800 ms and 800–1200 ms), to examine the temporal dynamics of this dependent variable (Figure 6B). The topographical distribution of this decrease in alpha power (Figure 6C) indicated that alpha-band power was reduced at temporal sites, in addition to the expected parieto-occipital locations. These outcomes demonstrate reduced alpha-band power at expected topographical sites following the onset of the tone, replicating robust findings for its involvement in attentional processing (Frey et al., 2014; Deng et al., 2019, 2020).

**FIGURE 7:**
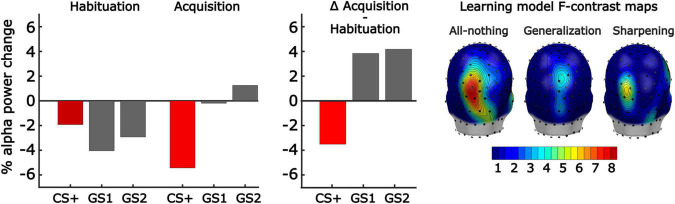
Effects of aversive generalization learning on stimulus-induced power changes in the alpha frequency band. **(Left)** Grand mean (*n* = 34) changes in alpha-band power, averaged across a posterior cluster of POz and its 5 nearest neighboring sensors, and across a time window of 300–800 ms post-stimulus (i.e., early time window). Note the strong alpha-band power reduction for the CS+ stimulus in acquisition, compared to habituation, indicative of learning effects. **(Middle)** The change in alpha-band power from habituation to acquisition for the three pitches is consistent with the All-or-Nothing learning model, reflecting discrimination learning to the GS1. **(Right)** Converging findings were seen in the mass univariate analysis of the early time window, submitting each sensor’s alpha-band power change score to fitting the linear contrast corresponding to each learning model. Most support was seen for the all-or-nothing discrimination model.

Results from our repeated measures ANOVA comparing differences in transient alpha-band power reduction in response to tone onset from acquisition to habituation yielded a main effect of Pitch, *F*(2,66) = 4.41, *p* = 0.016, $\eta_{\text{p}}^{2}$ = 0.14, BF_10_ = 3.79 (Figure 7). This main effect was examined using three *F*-contrast analyses performed for each learning model on the difference score in alpha-band power from habituation to acquisition, computed for each pitch. *F*-contrasts across the three values were then computed using the weights corresponding to the three competing learning models, as described above (Figure 7). Specifically, the following linear contrasts were observed for early window time points (i.e., 300–800 ms): generalization [*F*(1,68) = 3.3, *p* = 0.043], sharpening [*F*(1,68) = 1.6, *p* = 0.141], and all-or-nothing, *F*(1,68) = 6.3, *p* = 0.007. For late window time points (i.e., 800–1,200 ms) the following contrasts were observed: generalization [*F*(1,68) = 0.6, *p* = 0.379], sharpening [*F*(1,68) = 4.9, *p* = 0.016], and all-or-nothing, *F*(1,68) = 6.2, *p* = 0.008. These initial *F*-contrast tests suggested evidence for the all-or-nothing models across both early and late time windows of alpha-band power changes between habituation and acquisition. However, we conducted mass univariate analyses to ensure these outcomes were robust and not a product of the sensors or time points selected.

The mass univariate analyses for each time point and sensor, controlled by *F*_*max*_ permutation distributions, yielded converging results demonstrating strong evidence for the all-or-nothing learning model and weak evidence for the Generalization learning model in the early time window at parieto-occipital sites (Figure 7). Specifically, the permutation-controlled threshold was exceeded for the early time window (i.e., 300–800 ms) at three adjacent parieto-occipital sensors. We also observed strong evidence for the all-or-nothing model, and the sharpening model at the same sites in the later time window (i.e., 800–1,200 ms). None of the other model-based contrasts crossed the permutation-based threshold at any electrode or time point. Thus, changes in alpha-band power reduction were strongest for the CS+, and held a better fit with the all-or-nothing learning model.

**FIGURE 8:**
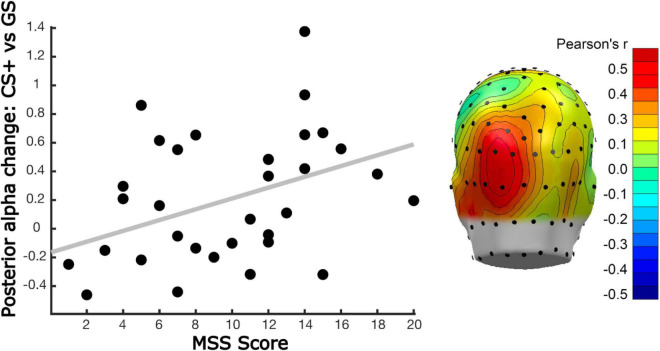
Linear relation between posterior alpha-band power changes and MSS. **(Left)** Scatter plot showing the relationship between the All-or-Nothing learning model (i.e., selective alpha-band power reduction for the CS+, with little to no change for the GS1 and GS2) fit with parieto-occipital alpha-band power changes, and correlated with the MSS score of each participant. Alpha-band power reduction was computed by averaging time-varying power changes (acquisition minus habituation) in a time window from 300 to 800 ms post-tone onset (i.e., early time window), across sensor POz, and its 5 nearest neighboring sensors. **(Right)** Mass univariate analysis of correlations between the All-or-Nothing model fit applied to alpha-band power changes and MSS scores, with Person’s *r*-values between these variables color coded. The results of this analysis converged with the window average analysis in the left panel. A cluster of posterior sensors (dark red) crossed the permutation-controlled threshold for statistical significance (*r* > 0.50), during an interval of 520 to 640 ms. The topographical distribution of this effect shows the mean correlation in that time window, following Fisher-*z* transformation, averaging across time points, and re-transformed to correlation coefficients.

**FIGURE 9:**
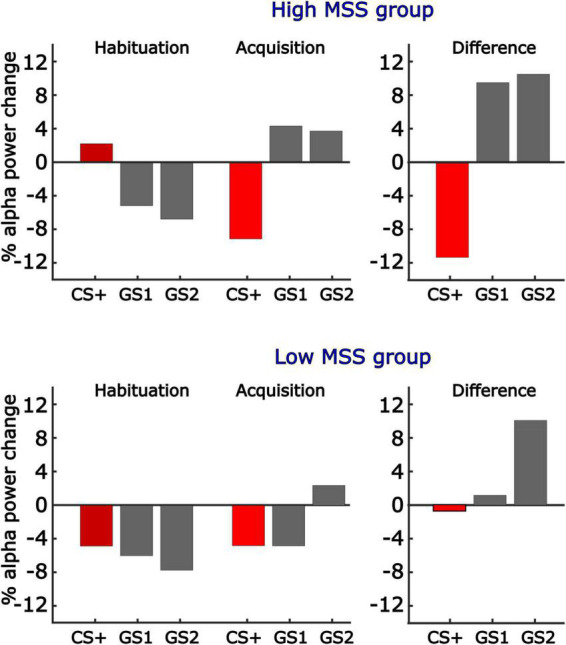
Illustration of differences in generalization learning as a function of MSS score. The present report considered MSS scores as a continuous variable. To illustrate the correlation between MSS scores and All-or-Nothing model fit, this figure shows the mean changes in alpha-band power, with learning-induced changes expressed as the difference score, for the 10 individuals with the highest and lowest MSS. Consistent with the positive correlation between the All-or-Nothing learning model fit of alpha-band power changes and MSS scores as a continuous variable, individuals with high self-reported Misophonia symptoms displayed strong discrimination learning, with no generalization or sharpening present. By contrast, participants in the low MSS group (i.e., 10 participants with lowest MSS scores), showed little learning effects and displayed some evidence of generalization.

Participants’ MSS scores were differentially associated with our learning models. This was observed by computing the inner product of each model with the corresponding alpha-band power differences, resulting in a single value per subject, electrode, and time point that reflected the fit of the respective model with our alpha-band power data. This value, for both early and late time windows, was then correlated across participants’ MSS scores obtained for averaged alpha-band power in early and late windows. This was also done in a mass-univariate fashion, for each sensor and time point. Again, the two analyses converged, showing that individuals with higher MSS scores showed more pronounced all-or-nothing learning model in the early time window, and that this correlation was greatest at parieto-occipital sites (Figure 8). Correlations were unaffected by co-varying out Hyperacusis thresholds, which were not associated with MSS scores in this sample, *r* = 0.03. No significant correlations between MSS and learning-induced changes were observed in the late time window in the mass-univariate analysis nor for the selected time and electrode averages. A subsequent exploratory analysis examining this linear relationship is further illustrated in Figure 9, in which we analyzed alpha-band power in two groups: those reporting the highest MSS and those with the lowest MSS (i.e., 10 per group). As in our continuous analyses, the high MSS group showed greater changes in alpha-band power reduction for the CS+, with their data fitting an all-or-nothing model stronger than those in the low MSS group. This suggests that individuals with endorsing higher scores on the MSS also exhibit stronger decreases in alpha-band power changes from habituation to acquisition phases in response to the CS+ versus the other GS conditions. More importantly, the all-or-nothing learning model was a stronger fit for these data in individuals with greater MSS.

## Discussion

The present preliminary report from an ongoing project aimed to identify the extent to which aversive generalization learning is systematically related to self-reported Misophonia symptoms. Pavlovian learning has long been hypothesized to be involved in the etiological nexus of Misophonia (Jastreboff and Jastreboff, 2001, 2015), as a cause or contributory factor. More recently, others have theorized that emotional responses to orofacial “trigger” sounds can be generalized to various environmental auditory stimuli (Dozier, 2015; Kumar et al., 2021; Vitoratou et al., 2021a) through associative learning. Because generalization learning has also yielded promising findings in fear and anxiety-related disorders (Lissek et al., 2005; Reinhardt et al., 2010; Duits et al., 2015), which share common symptomology with Misophonia (Ginsburg et al., 2006; Hadjipavlou et al., 2008; Edelstein et al., 2013; Wu et al., 2014; Dozier, 2015; Webber and Storch, 2015; Zhou et al., 2017; Quek et al., 2018; Erfanian et al., 2019; Potgieter et al., 2019; Jager et al., 2020; McKay and Acevedo, 2020), we examined the extent to which individuals differing in Misophonia symptoms varied in auditory aversive generalization learning. A classical conditioning approach was used to pair one of three initially neutral pitches of a sine-wave tone with a loud noise. Three dependent variables with known sensitivity to generalization learning were considered: self-reported valence and emotional arousal in response to each pitch (McTeague et al., 2015; Plog et al., 2022), as well as stimulus-induced reductions in parieto-occipital alpha power, a brain response linked to the attentive processing of aversively conditioned cues, auditory or visual (Miskovic and Keil, 2012; Yin et al., 2020; Friedl and Keil, 2021). Self-reported symptoms on the MSS were used to quantify the intensity of Misophonia symptoms in each participant.

Manipulation checks indicated that all dependent variables showed strong effects of the conditioning regimen, with selective responses to the CS+ apparent across the entire sample. Specifically, these effects were isolated to the acquisition phase, demonstrating participants learned the contingencies between each pitch of the sine-wave tone and the US. Comparing three prototypical models of generalization, we found that an all-or-nothing discrimination learning model was most pronounced across the sample, with little evidence for competing generalization and sharpened tuning models. All-or-nothing learning occurs when individuals respond selectively to the CS+, but do not respond differentially to the generalization stimuli, despite their similarities in physical characteristics (Friedl and Keil, 2020). As such, individuals responding in this pattern effectively identify and differentiate a stimulus based on specific attributes from other stimuli sharing similar properties.

For self-reported valence and arousal ratings, the CS+ elicited the most negative and arousing ratings, and this occurred primarily in the acquisition phase. Importantly, we also found strong evidence that heightened Misophonia symptoms are associated with more negative and greater arousal ratings for the sine-wave tone, regardless of pitch or the phase in which the tone was presented (i.e., habituation or acquisition) in the conditioning paradigm. This effect was not related to auditory tolerance threshold scores, determined through LDLs. However, auditory tolerance threshold scores did predict arousal, or intensity, ratings for the tone, regardless of pitch and experimental phase. Importantly, no interactions were found involving MSS. Thus, although MSS was strongly related to affective ratings of the tones at baseline, these findings suggest that the conditioning-induced change in affective ratings (i.e., valence and arousal) as well as the amount of generalization learning as reflected specifically in ratings did not vary as a function of Misophonia symptoms in the present sample.

Examination of the overall fit of our competing learning models to both valence and arousal data revealed that the strength of the models was not related to Misophonia symptoms, nor did the overall fit of any of our models vary based on MSS scores. Instead, we observed evidence supporting the all-or-nothing discrimination learning model, an effect that was mostly prominent in the acquisition phase. These analyses controlled for auditory tolerance threshold scores, which also were unrelated to model fit. These findings indicate that, regardless of the degree of Misophonia symptoms endorsed, individuals’ valence and arousal ratings were selectively higher for the CS+ compared to the other GS, and that responses to these latter stimuli were similarly low. Thus, participants were able to clearly distinguish the CS+ from other stimuli sharing similarities in auditory properties.

Parieto-occipital alpha-band power reduction has long been associated with responses to a salient external event, regardless of sensory modality (Berger, 1929; Friedl and Keil, 2021). Recently, these changes have been shown to index aversive conditioning, including generalization learning (Friedl and Keil, 2020; Yin et al., 2020). Consistent with the self-reported behavioral findings, we found that alpha-band power reduction showed pronounced activity patterns best fit by an all-or-nothing learning model across all participants at the predicted parieto-occipital regions where alpha power during rest is maximal. Specifically, the CS+ prompted pronounced alpha power reduction after, compared to before, conditioning, consistent with attentive processing. By contrast, both GS induced relative alpha power enhancement, consistent with reduced attention to these auditory cues. Quantifying the all-or-nothing pattern, along with two additional model-based patterns, showed the best fit of the all-or-nothing learning model both in a region-of-interest analysis and in a permutation-controlled mass univariate analysis. Neither the generalization nor sharpening learning models fit the empirical EEG data. Interestingly, MSS scores showed a strong positive linear relationship with the all-or-nothing learning pattern at parieto-occipital alpha locations, indicating that individuals endorsing Misophonia symptoms showed more pronounced discrimination learning. However, there was no evidence of generalization learning and limited evidence for sharpened tuning being associated with MSS scores. These results do not support the hypothesis that Misophonia is associated with heightened generalization (i.e., overgeneralization). Although overgeneralization may be present at other levels of analysis, such as sensory evoked responses or auditory cortical fMRI-BOLD, neither self-reported valence and arousal, nor alpha-band power reduction suggests that overgeneralization is related to Misophonia during a laboratory-based auditory conditioning regimen. In contrast, very strong linear relations were observed between MSS scores and affective ratings of valence and arousal, and this association was independent of the psychophysics-based proxy of hyperacusis used in the present study (i.e., auditory tolerance threshold). These main effects suggest that sine-wave tones, while tolerated, evoked greater self-reported feelings of aversive/defensive affect and arousal in those with Misophonia, regardless of their learned attributes and role in the conditioning paradigm. Such heightened aversive/defensive sensitivity in Misophonia has been discussed in the literature and has prompted discussions regarding the demarcation of Misophonia and hyperacusis or related conditions associated with sound aversion (Aazh et al., 2018; Jager et al., 2020).

At the level of parieto-occipital alpha-band power changes measured through scalp EEG, there was also strong evidence of an effect of MSS score, but this effect depended on the role a stimulus played in the generalization learning protocol. In contrast to the notion that Misophonia is associated with heightened generalization learning (overgeneralization), we observed heightened discrimination learning (all-or-nothing) in individuals endorsing high levels of Misophonia. However, Participants with lower MSS scores displayed less evidence of learning in their alpha-band power changes, and anecdotally displayed relatively heightened generalization compared to high MSS individuals. A larger sample is needed to characterize these differences, but they are consistent with the notion that individuals endorsing Misophonia symptoms display discriminating response patterns across a generalization gradient, such that they efficiently isolate an auditory CS+ from other similar stimuli sharing similar physical properties. In contrast, those without Misophonia may be less adept at being able to discriminate an auditory cue paired with a noxious event from other similar sounds.

Individuals with Misophonia endorse orofacial sounds as primary “trigger” cues (Jastreboff and Jastreboff, 2003, 2015; Edelstein et al., 2013; Schröder et al., 2013; Duddy and Oeding, 2014; Kumar et al., 2021; Vitoratou et al., 2021a; Swedo et al., 2022). In addition, others have proposed that these adverse emotional responses can generalize to other environmental sounds, which may not be orofacial in nature (Dozier, 2015; Kumar et al., 2021). Supporting this notion, Vitoratou et al. (2021a) found that individuals sensitive to orofacial sounds were also likely to have adverse reactions to environmental sounds, such as tapping keyboard or rustling paper. However, their results also indicated that a listener’s ability to discriminate “trigger” cues from other similar sounds largely relied upon individuals’ sound sensitivity. Specifically, environmental cues providing little information were clustered as having lower discrimination abilities (e.g., clocks, nails, etc.), such that they held lower sensitivity to be detected. In contrast, other sounds (e.g., car engine, rustling, and tapping) providing more information held greater sensitivity and were easier to discriminate. Taking these findings into account, it could be argued that the all-or-nothing discrimination model is most likely to occur for impoverished stimuli, such as pure tones. However, more research is required with different and more naturalistic auditory cues.

As noted above, the present study is limited by its preliminary nature owed to the still evolving sample. As such, several considerations should be taken regarding these outcomes. First, our sample size is limited (i.e., 34), and primarily consisted of undergraduate students from the University of Florida. As such, larger and more encompassing sample sizes may detect effects related to overgeneralization. In addition, studies with significantly larger sample sizes will be capable of appropriately co-varying other personality traits, such as neuroticism, that may have contributed to our findings. In a similar vein, averages scores on the MSS were 9.55, below Wu et al. (2014) recommended threshold for the presence of Misophonia. Given this, our sample was largely more non-misophonic. Third, our measurement of Misophonia symptomology was restricted to the use of the MSS subscale, which may not have as psychometrically sound as other Misophonia measures (Siepsiak et al., 2020; Rosenthal et al., 2021; Vitoratou et al., 2021b). For example, the MSS only assesses seven symptoms of Misophonia. In contrast, the Duke Misophonia Questionnaire measures several features of Misophonia, including affective, physiological, and cognitive symptoms in respective subscales.

Several conceptual limitations should also be considered regarding these preliminary results. Although we did not observe evidence of generalization mechanisms in Misophonia for the outcome measures reported on here, indices of other physiological processes involved in Pavlovian learning may well indicate generalization, paralleling a plethora of studies in aversive conditioning research (Hamm and Weike, 2005). It may also be the case that although generalization learning was not observed here, this does not rule out Pavlovian processes as an etiological mechanism in Misophonia. In addition, our study did not include formal auditory evaluations of participants using an audiologist, and only included assessment of LDLs. Thus, we may not have fully captured all dimensions of hyperacusis, and we were unable to rule out individuals experiencing tinnitus. In addition, future analyses will be able to examine additional variables, such as pupil diameter change, auditory steady-state responses, and fMRI BOLD during auditory aversive generalization learning. The results of such work will be in a better, more adequately powered, position to give a more complete picture of the robustness of the effects observed here, as well as examine the potential usefulness and psychometric properties of indices of aversive generalization learning for characterizing Misophonia.

## Data Availability Statement

The raw data supporting the conclusions of this article will be made available by the authors, without undue reservation.

## Ethics Statement

The studies involving human participants were reviewed and approved by University of Florida – Institutional Review Board. The patients/participants provided their written informed consent to participate in this study.

## Author Contributions

RW, FG, JP, PC, and SM contributed to data collection, analyses, manuscript drafting, and supervised the project. CT contributed to data collection and manuscript editing. KR contributed to manuscript editing. RM and AK contributed to study design, manuscript development, and analyses. All authors contributed to the article and approved the submitted version.

## Conflict of Interest

The authors declare that the research was conducted in the absence of any commercial or financial relationships that could be construed as a potential conflict of interest.

## Publisher’s Note

All claims expressed in this article are solely those of the authors and do not necessarily represent those of their affiliated organizations, or those of the publisher, the editors and the reviewers. Any product that may be evaluated in this article, or claim that may be made by its manufacturer, is not guaranteed or endorsed by the publisher.

## Footnotes

1
https://osf.io/e26ad
