# Supplementary material for: The Relationship Between Self-Reported Misophonia Symptoms and Auditory Aversive Generalization Leaning: A Preliminary Report
Source: Front Neurosci. 2022 Jun 23;16:899476. doi: 10.3389/fnins.2022.899476 (PMC9260228; doi:10.3389/fnins.2022.899476)
Supplement: Supplementary file 1 [file Data_Sheet_1.docx]

**APPENDIX A**

**Table 1. Misophonia Symptom Survey (MSS)**

Do the following sounds bother you more than they bother other people you know?

| Items | Never | Rarely | Sometimes | Often | Always |
| --- | --- | --- | --- | --- | --- |
| 1. People eating (e.g., chewing, swallowing, lips smacking, slurping, etc.) | 〇 | 〇 | 〇 | 〇 | 〇 |
| 2. Repetitive tapping (e.g., pen on table, foot on floor, etc.) | 〇 | 〇 | 〇 | 〇 | 〇 |
| 3. Rustling (e.g., plastic, paper, etc.) | 〇 | 〇 | 〇 | 〇 | 〇 |
| 4. People making nasal sounds (e.g., inhale, exhale, sniffing, etc.) | 〇 | 〇 | 〇 | 〇 | 〇 |
| 5. People making throat sounds (e.g., throat clearing, coughing, etc.) | 〇 | 〇 | 〇 | 〇 | 〇 |
| 6. Certain consonants and/or vowels (e.g. "k" sounds, etc.) | 〇 | 〇 | 〇 | 〇 | 〇 |
| 7. Environmental sounds (e.g., clock ticking, refrigerator humming, etc.) | 〇 | 〇 | 〇 | 〇 | 〇 |

**Note:** Responses scored from 0 (i..e, Never) to 4 (i.e., Always). Total scores range from 0 to 28

**Table 2. Raw Behavioral Interaction Contrasts**

|  | Valence | | |  | Arousal | | |
| --- | --- | --- | --- | --- | --- | --- | --- |
| Contrast | *t* value | df | *p* value |  | *t* value | df | *p* value |
| CS+ Early Habituation – GS1 Early Habituation | -0.39 | 385 | > 0.999 |  | 1.88 | 385 | > 0.999 |
| CS+ Early Habituation –GS2 Early Habituation | -1.05 | 385 | > 0.999 |  | -0.27 | 385 | > 0.999 |
| CS+ Early Habituation – CS+ Late Habituation | -0.09 | 385 | > 0.999 |  | 1.91 | 385 | > 0.999 |
| CS+ Early Habituation – GS1 Late Habituation | -1.59 | 385 | > 0.999 |  | 0.40 | 385 | > 0.999 |
| CS+ Early Habituation – GS2 Late Habituation | -2.28 | 385 | > 0.999 |  | -1.07 | 385 | > 0.999 |
| CS+ Early Habituation – CS+ Early Acquisition | -9.66 | 385 | **< 0.001***** |  | -11.91 | 385 | **< 0.001***** |
| CS+ Early Habituation – GS1 Early Acquisition | -0.20 | 385 | > 0.999 |  | 0.17 | 385 | > 0.999 |
| CS+ Early Habituation – GS2 Early Acquisition | -1.04 | 385 | > 0.999 |  | 0.09 | 385 | > 0.999 |
| CS+ Early Habituation – CS+ Late Acquisition | -7.61 | 385 | **< 0.001***** |  | -9.06 | 385 | **< 0.001***** |
| CS+ Early Habituation – GS1 Late Acquisition | -1.46 | 385 | > 0.999 |  | -1.49 | 385 | > 0.999 |
| CS+ Early Habituation – GS2 Late Acquisition | -1.47 | 385 | > 0.999 |  | -0.75 | 385 | > 0.999 |
| CS+ Late Habituation – GS1 Late Habituation | -1.69 | 385 | > 0.999 |  | -1.51 | 385 | > 0.999 |
| CS+ Late Habituation – GS2 Late Habituation | -2.38 | 385 | > 0.999 |  | -2.58 | 385 | 0.201 |
| CS+ Late Habituation – CS+ Early Acquisition | -9.75 | 385 | **< 0.001***** |  | -13.82 | 385 | **< 0.001***** |
| CS+ Late Habituation – GS1 Early Acquisition | -0.29 | 385 | > 0.999 |  | -1.74 | 385 | > 0.999 |
| CS+ Late Habituation – GS2 Early Acquisition | -1.13 | 385 | > 0.999 |  | -1.82 | 385 | > 0.999 |
| CS+ Late Habituation – CS+ Late Acquisition | -7.70 | 385 | **< 0.001***** |  | -10.97 | 385 | **< 0.001***** |
| CS+ Late Habituation – GS1 Late Acquisition | -1.55 | 385 | > 0.999 |  | -3.39 | 385 | 0.050 |
| CS+ Late Habituation – GS2 Late Acquisition | -1.56 | 385 | > 0.999 |  | -2.66 | 385 | 0.542 |
| CS+ Early Acquisition – GS1 Early Acquisition | 9.46 | 385 | **< 0.001***** |  | 12.08 | 385 | **< 0.001***** |
| CS+ Early Acquisition – GS2 Early Acquisition | 8.62 | 385 | **< 0.001***** |  | 12.00 | 385 | **< 0.001***** |
| CS+ Early Acquisition – CS+ Late Acquisition | 2.05 | 385 | > 0.999 |  | 2.85 | 385 | 0.305 |
| CS+ Early Acquisition – GS1 Late Acquisition | 8.20 | 385 | **< 0.001***** |  | 10.42 | 385 | **< 0.001***** |
| CS+ Early Acquisition – GS2 Late Acquisition | 8.19 | 385 | **< 0.001***** |  | 11.16 | 385 | **< 0.001***** |
| CS+ Late Acquisition – GS1 Late Acquisition | 6.15 | 385 | **< 0.001***** |  | 7.57 | 385 | **< 0.001***** |
| CS+ Late Acquisition – GS2 Late Acquisition | 6.14 | 385 | **< 0.001***** |  | 8.31 | 385 | **< 0.001***** |
| GS1 Early Habituation – GS2 Early Habituation | -0.67 | 385 | > 0.999 |  | -2.15 | 385 | > 0.999 |
| GS1 Early Habituation – CS+ Late Habituation | 0.48 | 385 | > 0.999 |  | 0.03 | 385 | > 0.999 |
| GS1 Early Habituation – GS1 Late Habituation | -1.21 | 385 | > 0.999 |  | -1.48 | 385 | > 0.999 |
| GS1 Early Habituation – GS2 Late Habituation | -1.90 | 385 | > 0.999 |  | -2.95 | 385 | 0.221 |
| GS1 Early Habituation – CS+ Early Acquisition | -9.27 | 385 | **< 0.001***** |  | -13.78 | 385 | **< 0.001***** |
| GS1 Early Habituation – GS1 Early Acquisition | 0.19 | 385 | > 0.999 |  | -1.71 | 385 | > 0.999 |
| GS1 Early Habituation – GS2 Early Acquisition | -0.65 | 385 | > 0.999 |  | -1.79 | 385 | > 0.999 |
| GS1 Early Habituation – CS+ Late Acquisition | -7.22 | 385 | **< 0.001***** |  | -10.94 | 385 | **< 0.001***** |
| GS1 Early Habituation – GS1 Late Acquisition | -1.07 | 385 | > 0.999 |  | -3.36 | 385 | 0.056 |
| GS1 Early Habituation – GS2 Late Acquisition | -1.08 | 385 | > 0.999 |  | -2.63 | 385 | 0.593 |
| GS1 Late Habituation – GS2 Late Habituation | -0.69 | 385 | > 0.999 |  | -1.47 | 385 | > 0.999 |
| GS1 Late Habituation – CS+ Early Acquisition | -8.07 | 385 | **< 0.001***** |  | -12.31 | 385 | **< 0.001***** |
| GS1 Late Habituation – GS1 Early Acquisition | 1.40 | 385 | > 0.999 |  | -0.23 | 385 | > 0.999 |
| GS1 Late Habituation – GS2 Early Acquisition | 0.55 | 385 | > 0.999 |  | -0.31 | 385 | > 0.999 |
| GS1 Late Habituation – CS+ Late Acquisition | -6.02 | 385 | **< 0.001***** |  | -9.46 | 385 | **< 0.001***** |
| GS1 Late Habituation – GS1 Late Acquisition | 0.13 | 385 | > 0.999 |  | -1.89 | 385 | > 0.999 |
| GS1 Late Habituation – GS2 Late Acquisition | 0.12 | 385 | > 0.999 |  | -1.15 | 385 | > 0.999 |
| GS1 Early Acquisition – GS2 Early Acquisition | -0.84 | 385 | > 0.999 |  | -0.08 | 385 | > 0.999 |
| GS1 Early Acquisition – CS+ Late Acquisition | -7.41 | 385 | **< 0.001***** |  | -9.23 | 385 | **< 0.001***** |
| GS1 Early Acquisition – GS1 Late Acquisition | -1.26 | 385 | > 0.999 |  | -1.66 | 385 | > 0.999 |
| GS1 Early Acquisition – GS2 Late Acquisition | -1.27 | 385 | > 0.999 |  | -0.92 | 385 | > 0.999 |
| GS1 Late Acquisition – GS2 Late Acquisition | -0.01 | 385 | > 0.999 |  | 0.74 | 385 | > 0.999 |
| GS2 Early Habituation – CS+ Late Habituation | 1.15 | 385 | > 0.999 |  | 2.18 | 385 | > 0.999 |
| GS2 Early Habituation – GS1 Late Habituation | -0.54 | 385 | > 0.999 |  | 0.67 | 385 | > 0.999 |
| GS2 Early Habituation – GS2 Late Habituation | -1.23 | 385 | > 0.999 |  | -0.80 | 385 | > 0.999 |
| GS2 Early Habituation – CS+ Early Acquisition | -8.61 | 385 | **< 0.001***** |  | -11.63 | 385 | **< 0.001***** |
| GS2 Early Habituation – GS1 Early Acquisition | 0.86 | 385 | > 0.999 |  | 0.45 | 385 | > 0.999 |
| GS2 Early Habituation – GS2 Early Acquisition | 0.02 | 385 | > 0.999 |  | 0.37 | 385 | > 0.999 |
| GS2 Early Habituation – CS+ Late Acquisition | -6.55 | 385 | **< 0.001***** |  | -8.78 | 385 | **< 0.001***** |
| GS2 Early Habituation – GS1 Late Acquisition | -0.40 | 385 | > 0.999 |  | -1.21 | 385 | > 0.999 |
| GS2 Early Habituation – GS2 Late Acquisition | -0.42 | 385 | > 0.999 |  | -0.47 | 385 | > 0.999 |
| GS2 Late Habituation – CS+ Early Acquisition | -7.38 | 385 | **< 0.001***** |  | -10.83 | 385 | **< 0.001***** |
| GS2 Late Habituation – GS1 Early Acquisition | 2.09 | 385 | > 0.999 |  | 1.25 | 385 | > 0.999 |
| GS2 Late Habituation – GS2 Early Acquisition | 1.24 | 385 | > 0.999 |  | 1.17 | 385 | > 0.999 |
| GS2 Late Habituation – CS+ Late Acquisition | -5.32 | 385 | **< 0.001***** |  | -7.98 | 385 | **< 0.001***** |
| GS2 Late Habituation – GS1 Late Acquisition | 0.83 | 385 | > 0.999 |  | -0.41 | 385 | > 0.999 |
| GS2 Late Habituation – GS2 Late Acquisition | 0.81 | 385 | > 0.999 |  | 0.33 | 385 | > 0.999 |
| GS2 Early Acquisition – CS+ Late Acquisition | -6.57 | 385 | **< 0.001***** |  | -9.15 | 385 | **< 0.001***** |
| GS2 Early Acquisition – GS1 Late Acquisition | -0.42 | 385 | > 0.999 |  | -1.58 | 385 | > 0.999 |
| GS2 Early Acquisition – GS2 Late Acquisition | -0.43 | 385 | > 0.999 |  | -0.84 | 385 | > 0.999 |

**Note:** All reported *p* values are Bonferroni corrected. Bold *p* values indicate significant model comparisons.

****p* < 0.001.

**Table 3. Model Behavioral Interaction Contrasts**

|  | Valence | | |  | Arousal | | | |  |
| --- | --- | --- | --- | --- | --- | --- | --- | --- | --- |
| Contrast | *t* value | df | *p* value |  | | *t* value | df | *p* value | |
| Generalization Early Habituation – Sharpening Early Habituation | -0.84 | 385 | > 0.999 |  | | -2.58 | 385 | 0.6690 | |
| Generalization Early Habituation –All-or-Nothing Early Habituation | -0.06 | 385 | > 0.999 |  | | -1.86 | 385 | > 0.999 | |
| Generalization Early Habituation – Generalization Late Habituation | 0.75 | 385 | > 0.999 |  | | 118 | 385 | > 0.999 | |
| Generalization Early Habituation – Sharpening Late Habituation | -0.12 | 385 | > 0.999 |  | | -0.60 | 385 | > 0.999 | |
| Generalization Early Habituation – All-or-Nothing Late Habituation | 1.41 | 385 | > 0.999 |  | | 1.39 | 385 | > 0.999 | |
| Generalization Early Habituation – Generalization Early Acquisition | -5.36 | 385 | **< 0.001***** |  | | -7.38 | 385 | **< 0.001***** | |
| Generalization Early Habituation – Sharpening Early Acquisition | -6.42 | 385 | **< 0.001***** |  | | -7.48 | 385 | **< 0.001***** | |
| Generalization Early Habituation – All-or-Nothing Early Acquisition | -11.02 | 385 | **< 0.001***** |  | | -13.86 | 385 | **< 0.001***** | |
| Generalization Early Habituation – Generalization Late Acquisition | -4.31 | 385 | 0.0014 |  | | -5.74 | 385 | **< 0.001***** | |
| Generalization Early Habituation – Sharpening Late Acquisition | -4.33 | 385 | 0.0013 |  | | -4.85 | 385 | 0.0001 | |
| Generalization Early Habituation – All-or-Nothing Late Acquisition | -7.77 | 385 | **< 0.001***** |  | | -9.49 | 385 | **< 0.001***** | |
| Generalization Late Habituation – Sharpening Late Habituation | -0.87 | 385 | > 0.999 |  | | -1.77 | 385 | > 0.999 | |
| Generalization Late Habituation – All-or-Nothing Late Habituation | 0.65 | 385 | > 0.999 |  | | 0.215 | 385 | > 0.999 | |
| Generalization Late Habituation – Generalization Early Acquisition | -6.12 | 385 | **< 0.001***** |  | | -8.56 | 385 | **< 0.001***** | |
| Generalization Late Habituation – Sharpening Early Acquisition | -7.17 | 385 | **< 0.001***** |  | | -8.65 | 385 | **< 0.001***** | |
| Generalization Late Habituation – All-or-Nothing Early Acquisition | -11.77 | 385 | **< 0.001***** |  | | -15.03 | 385 | **< 0.001***** | |
| Generalization Late Habituation – Generalization Late Acquisition | -5.06 | 385 | **< 0.001***** |  | | -6.91 | 385 | **< 0.001***** | |
| Generalization Late Habituation – Sharpening Late Acquisition | -5.08 | 385 | **< 0.001***** |  | | -6.03 | 385 | **< 0.001***** | |
| Generalization Late Habituation – All-or-Nothing Late Acquisition | -8.52 | 385 | **< 0.001***** |  | | -10.66 | 385 | **< 0.001***** | |
| Generalization Early Acquisition – Sharpening Early Acquisition | -1.06 | 385 | > 0.999 |  | | -0.10 | 385 | > 0.999 | |
| Generalization Early Acquisition – All-or-Nothing Early Acquisition | -5.66 | 385 | **< 0.001***** |  | | -6.48 | 385 | **< 0.001***** | |
| Generalization Early Acquisition – Generalization Late Acquisition | 1.04 | 385 | > 0.999 |  | | 1.64 | 385 | > 0.999 | |
| Generalization Early Acquisition – Sharpening Late Acquisition | 1.03 | 385 | > 0.999 |  | | 2.53 | 385 | 0.7851 | |
| Generalization Early Acquisition – All-or-Nothing Late Acquisition | -2.41 | 385 | > 0.999 |  | | -2.11 | 385 | > 0.999 | |
| Generalization Late Acquisition – Sharpening Late Acquisition | -0.01 | 385 | > 0.999 |  | | 0.89 | 385 | > 0.999 | |
| Generalization Late Acquisition – All-or-Nothing Late Acquisition | 0.04 | 385 | 0.0404 |  | | -3.75 | 385 | 0.0136 | |
| Sharpening Early Habituation – All-or-Nothing Early Habituation | 0.78 | 385 | > 0.999 |  | | 0.72 | 385 | > 0.999 | |
| Sharpening Early Habituation – Generalization Late Habituation | 1.59 | 385 | > 0.999 |  | | 3.76 | 385 | 0.0130 | |
| Sharpening Early Habituation – Sharpening Late Habituation | 0.72 | 385 | > 0.999 |  | | 1.99 | 385 | > 0.999 | |
| Sharpening Early Habituation – All-or-Nothing Late Habituation | 2.25 | 385 | > 0.999 |  | | 3.97 | 385 | 0.0056 | |
| Sharpening Early Habituation – Generalization Early Acquisition | -4.51 | 385 | 0.0006 |  | | -4.80 | 385 | 0.0002 | |
| Sharpening Early Habituation – Sharpening Early Acquisition | -5.58 | 385 | **< 0.001***** |  | | -4.89 | 385 | 0.0001 | |
| Sharpening Early Habituation – All-or-Nothing Early Acquisition | -10.18 | 385 | **< 0.001***** |  | | -11.28 | 385 | **< 0.001***** | |
| Sharpening Early Habituation – Generalization Late Acquisition | -3.47 | 385 | 0.0384 |  | | -3.15 | 385 | 0.1146 | |
| Sharpening Early Habituation – Sharpening Late Acquisition | -3.48 | 385 | 0.0365 |  | | -2.27 | 385 | > 0.999 | |
| Sharpening Early Habituation – All-or-Nothing Late Acquisition | -6.92 | 385 | **< 0.001***** |  | | -6.90 | 385 | **< 0.001***** | |
| Sharpening Late Habituation – All-or-Nothing Late Habituation | 1.53 | 385 | > 0.999 |  | | 1.99 | 385 | > 0.999 | |
| Sharpening Late Habituation – Generalization Early Acquisition | -5.23 | 385 | **< 0.001***** |  | | -6.78 | 385 | **< 0.001***** | |
| Sharpening Late Habituation – Sharpening Early Acquisition | -6.30 | 385 | **< 0.001***** |  | | -6.88 | 385 | **< 0.001***** | |
| Sharpening Late Habituation – All-or-Nothing Early Acquisition | -10.90 | 385 | **< 0.001***** |  | | -13.26 | 385 | **< 0.001***** | |
| Sharpening Late Habituation – Generalization Late Acquisition | -4.19 | 385 | 0.0023 |  | | -5.14 | 385 | **< 0.001***** | |
| Sharpening Late Habituation – Sharpening Late Acquisition | -4.21 | 385 | 0.0021 |  | | -4.26 | 385 | 0.0017 | |
| Sharpening Late Habituation – All-or-Nothing Late Acquisition | -7.65 | 385 | **< 0.001***** |  | | -8.89 | 385 | **< 0.001***** | |
| Sharpening Early Acquisition – All-or-Nothing Early Acquisition | -4.60 | 385 | 0.0004 |  | | -6.38 | 385 | **< 0.001***** | |
| Sharpening Early Acquisition – Generalization Late Acquisition | 2.12 | 385 | > 0.999 |  | | 1.74 | 385 | > 0.999 | |
| Sharpening Early Acquisition – Sharpening Late Acquisition | 2.09 | 385 | > 0.999 |  | | 2.62 | 385 | 0.5969 | |
| Sharpening Early Acquisition – All-or-Nothing Late Acquisition | -1.35 | 385 | > 0.999 |  | | -2.01 | 385 | > 0.999 | |
| Sharpening Late Acquisition – All-or-Nothing Late Acquisition | -3.44 | 385 | 0.0424 |  | | -4.63 | 385 | 0.0003 | |
| All-or-Nothing Early Habituation – Generalization Late Habituation | 0.82 | 385 | > 0.999 |  | | 3.04 | 385 | 0.1669 | |
| All-or-Nothing Early Habituation – Sharpening Late Habituation | -0.06 | 385 | > 0.999 |  | | 1.27 | 385 | > 0.999 | |
| All-or-Nothing Early Habituation – All-or-Nothing Late Habituation | 1.47 | 385 | > 0.999 |  | | 3.26 | 385 | 0.0815 | |
| All-or-Nothing Early Habituation – Generalization Early Acquisition | -5.29 | 385 | **< 0.001***** |  | | -5.52 | 385 | **< 0.001***** | |
| All-or-Nothing Early Habituation – Sharpening Early Acquisition | -6.36 | 385 | **< 0.001***** |  | | -5.61 | 385 | **< 0.001***** | |
| All-or-Nothing Early Habituation – All-or-Nothing Early Acquisition | -10.96 | 385 | **< 0.001***** |  | | -11.99 | 385 | **< 0.001***** | |
| All-or-Nothing Early Habituation – Generalization Late Acquisition | -4.25 | 385 | 0.0018 |  | | -3.87 | 385 | 0.0083 | |
| All-or-Nothing Early Habituation – Sharpening Late Acquisition | -4.26 | 385 | 0.0017 |  | | -2.99 | 385 | 0.1974 | |
| All-or-Nothing Early Habituation – All-or-Nothing Late | -7.70 | 385 | **< 0.001***** |  | | -7.62 | 385 | **< 0.001***** | |
| All-or-Nothing Late Habituation – Generalization Early Acquisition | -6.76 | 385 | **< 0.001***** |  | | -8.77 | 385 | **< 0.001***** | |
| All-or-Nothing Late Habituation – Sharpening Early Acquisition | -7.83 | 385 | **< 0.001***** |  | | -8.87 | 385 | **< 0.001***** | |
| All-or-Nothing Late Habituation – All-or-Nothing Early Acquisition | -12.43 | 385 | **< 0.001***** |  | | -15.25 | 385 | **< 0.001***** | |
| All-or-Nothing Late Habituation – Generalization Late Acquisition | -5.72 | 385 | **< 0.001***** |  | | -7.13 | 385 | **< 0.001***** | |
| All-or-Nothing Late Habituation – Sharpening Late Acquisition | -5.73 | 385 | **< 0.001***** |  | | -6.24 | 385 | **< 0.001***** | |
| All-or-Nothing Late Habituation – All-or-Nothing Late Acquisition | -9.17 | 385 | **< 0.001***** |  | | -10.88 | 385 | **< 0.001***** | |
| All-or-Nothing Early Acquisition – Generalization Late Acquisition | 6.71 | 385 | **< 0.001***** |  | | 8.12 | 385 | **< 0.001***** | |
| All-or-Nothing Early Acquisition – Sharpening Late Acquisition | 6.69 | 385 | **< 0.001***** |  | | 9.01 | 385 | **< 0.001***** | |
| All-or-Nothing Early Acquisition – All-or-Nothing Late Acquisition | 3.25 | 385 | 0.0820 |  | | 4.37 | 385 | 0.0010 | |

**Note:** All reported *p* values are Bonferroni corrected. Bold *p* values indicate significant model comparisons.

****p* < 0.001.
